# Preference for complementary and alternative medicine among patients on long-term treatment in Jos university teaching hospital, Nigeria

**DOI:** 10.4103/jomt.jomt_29_20

**Published:** 2020-09-11

**Authors:** Tolulope O. Afolaranmi, Zuwaira I. Hassan

**Affiliations:** 1 Department of Community Medicine, University of Jos and Jos University Teaching Hospital, Jos Plateau State, Nigeria; 2 Department of Community Medicine, Abubakar Tafawa Balewa University, Bauchi, Bauchi State, Nigeria

**Keywords:** Complementary and alternative medicine, long-term treatment, preference, predictors, Nigeria

## Abstract

**Background:**

The use of complementary and alternative medicines(CAM) is on the increase globally particularly among those with chronic medical conditions. Imperatively, the treatment outcomes of management of chronic illness is hinged on adherence to prescribed conventional treatment with little or no attention paid to the intent to use or concomitant use of alternative medicines in most treatment settings. Hence, this study assessed the preference for CAM and its predictors as among patients on long-term treatment in Jos University Teaching Hospital.

**Methods:**

This was a cross-sectional study conducted among 176 patients accessing treatment for chronic medical conditions in Jos University Teaching Hospital using quantitative method of data collection. Epi Info statistical software version 7 was used for data analysis with odds ratio and 95% confidence interval used as point and interval estimates respectively while a *P*-value of <0.05 was considered statistically significant.

**Results:**

The median age of respondents was 50 (IQR 30–84) years with 83 (47.2%) being 51 years and above. Preference for CAM was reported by 26 (14.8%) with absence of side effects (AOR = 11.3; 95% CI= 5.8299–15.1185) being the sole predictor of preference for CAM.

**Conclusion:**

This study has demonstrated some level of preference for CAM among patients on long term conventional treatment with perceived absence of side effects influencing this preference level.

## INTRODUCTION

Alternative Medicines (AM) are practices and products not currently considered integral parts of conventional medicine but when used as adjunct to conventional medical treatment, are considered Complementary and Alternative Medicines (CAM).^[1–5]^ The use of CAM is on the increase globally particularly among those with chronic medical conditions with findings from studies conducted in developed countries documenting its use between 30% to 50% in adult populations.^[1,6–9]^ However, limited studies have documented the level of preference CAM and its attending factors particularly in resource-limited settings as Nigeria.^[8,9]^ Globally, chronic medical conditions account for more than 36 million deaths annually with low- and middle-income countries accounting for about 80% of these deaths.^[10,11]^ imperatively, the treatment outcomes of management of chronic illness are hinged on adherence to prescribed conventional treatment with little or no attention paid to the intent to use or concomitant use of alternative medicines in most treatment settings.^[2]^ Hence, this study was conducted to assess the preference for complementary and alternative medicines and its predictors as among patients on long term treatment in Jos University Teaching Hospital.

## MATERIALS AND METHODS

### Study setting

This study was conducted at the cardiology, endocrinology, hematology, nephrology, oncology, gastroenterology, pulmonology and rheumatology out-patient clinics of Jos University Teaching Hospital (JUTH). JUTH is one of the tertiary health institutions in Plateau state, North central Nigeria with an estimated bed capacity of 600 located in the Lamingo area of Jos North Local Government.^[12]^

### Study population

The study population comprised of all adult patients attending cardiology, endocrinology, hematology, nephrology, oncology, gastroenterology, pulmonology and rheumatology out-patient clinics in Jos University Teaching Hospital, Jos for at least one year prior to the time of the study.

### Study design

This was a cross-sectional study design assessing the preference for CAM and its predictors using quantitative method of data collection.

### Sample size estimation

The sample size for this study was determined using the appropriate sample size estimation formula for a cross-sectional study.^[13]^Where *n* is the minimum sample size, Z is the standard normal deviate at 95% confidence interval (1.96), q is the complementary probability (1–*P*), d is the precision of the study set at 0.05 and p is the preference for CAM from a previous study (87.7%).^[14]^ This gave a sample size of 176 after adjusting for poor and or incomplete responses.

### Criteria for inclusion in the study

All adult patients who had been attending their respective clinics for one year upwards, who were registered and booked for appointments within two months prior to the study were eligible for inclusion in the study. While patients with visual and speech impairment that will require a third-party involvement were excluded from the study. This was done to ensure that information obtained was volunteered by the patients and as such representative of the patient’s personal opinions within the context of the study objective. One year was used as a cut off for inclusion in line with the definition of chronic diseases adopted for this study^[15]^ as well as to have enabled the patients to have sufficient interaction with the health care system. Chronic diseases are conditions that last 1 year or more and require ongoing medical attention or limited activities of daily living or both.^[15]^

### Sampling technique

A stratified sampling technique was used in view of the fact that the respective out-patient clinics had varying number of booked patients. A list of all the patients who had met the inclusion criteria was obtained forming the sampling frame after abstracting their details from their respective clinic’s monthly booking registers followed by the review of their hospital records for ascertaining the fulfilment of the inclusion criteria. Following which proportion to size technique was used to obtain the number of patients to be sampled from each of the clinics. This was done by dividing the number of patients who had met the inclusion criteria booked for each clinic for a two month period prior to the study (cardiology – 129, endocrinology – 154, hematology – 88, nephrology – 80, oncology – 14, gastroenterology – 133, pulmonology – 57 and rheumatology – 42) by total number of patients who met inclusion criteria booked for all the clinics (697) multiplied by the sample size of 176. This gave the following number per clinic: cardiology – 38, endocrinology – 33, hematology – 22, nephrology – 20, oncology – 4, gastroenterology – 34, pulmonology – 14 and rheumatology – 11. Thereafter, the sampling frame for each clinic was used to allocate numbers to all the patients in ascending order from which computer-generated table of random numbers using WINPEPI statistical software for epidemiologist version 11.44 (J.H Abramson®) was used to select determined number of patients for each clinic respectively without replacement. These patients were then sampled on their respective clinic days for a period of two months. For the patients who declined consent for participation, repeat selections using the table of random numbers per clinic was done until the sample size was met.

### Data collection instrument

A semi-structured, interviewer-administered questionnaire adapted from previous studies following literature review was used to collect data from the respondents on sociodemographic characteristics, nature and duration of illness, utilization of CAM and factors influencing preference for CAM.^[1,8,16,17]^ The questionnaire was translated to Hausa, which was the language understood by most of the respondents and back translated to English language to ensure that its content was retained and preserved. Three research assistants were trained on the content and method of administration of questionnaire prior to the commencement of the study by the principal researcher. The data collection instrument was pretested among 10% of the estimated sample size at Plateau Specialist hospital among similar patients to address ambiguity in the questions, estimate administration time for proper planning for data collection and assess appropriateness of the contents in addressing the objectives of the study.

### Data collection procedure

Data were collected using a paper-based semi-structured, interviewer-administered questionnaire. Three trained research assistants administered the questionnaires to study participants immediately after their respective clinic consultations using the individual patient’s hospital numbers as identifiers.

### Ethical approval

Ethical clearance was obtained from Jos University Teaching Hospital Institutional Health Research Ethical Committee (JUTH/DCS/ADM/127/XXIX/1394). Written and verbal informed consents were obtained from all the respondents with confidentiality and anonymity of their responses assured and maintained.

### Data analysis

The data obtained were processed and analyzed using Epi info statistical software version 7(CDC, Atlanta GA) Demographic characteristics of the respondents such as age, sex, marital status, employment status, level of education, place of residence etc., duration of illness, co-morbidity status, and reasons for preference of CAM were all categorized as explanatory variables while the outcome variable was the preference for CAM assessed as yes or no. Characteristics of the respondents that are attributory in nature such as age group, sex, level of education, marital status, employment status, co-morbidity status etc. were expressed in frequency and percentage. Median and interquartile range were used as summary indices for numeric variables such age of the respondents and duration of illness having demonstrated skewness. A two-step approach to logistic regression was employed in identifying the predictors of preference for CAM. Binary logistic regression was conducted by feeding each of the characteristics of the respondents designated as explanatory variables singly in the binary model following which those with probability values of < 0.05 while then fed cumulative in to the multiple logistic regression models as the second step. Crude and adjusted odds ratios as well as 95% confidence interval were used as point and interval estimates of the effects of these factors on preference for CAM while a probability value of less than 0.05 was considered statistically significant.

## RESULTS

The median age of study participants was 50 (IQR30–84) years with 83 (47.2%) being 51 years and above. Furthermore, 108 (61.4%) of the respondents were females and most (73.9%) of them were married. With regards to highest level of education attained, 95 (54.0%) had attained tertiary level of education while 98 (55.7%) were engaged in paid jobs at the time of the study. In the study, 81 (46.0%) of the participants had been diagnosed of their medical conditions for more than 6 years with 48 (27.3%) having co-morbid conditions. Preference for CAM was reported by 26 (14.8%) of the respondents while absence of side effects, congruence with culture and beliefs and well as accessibility were mentioned as reasons for preference of CAM by 24 (92.3%), 21 (80.8%) and 22 (84.6%) of the study participants respectively [Table 1].

Preference for alternative medicines was found to be significantly influenced by perceived absence of side effects with the odds of its preference among those who adjudged it as having little or no side effects being 11.3 times (95% CI: 5.8299–15.1185) those who attributed side effects to it particularly in comparison to the convention medications after adjusting for other factors such as affordability, accessibility and congruence with culture and beliefs [Table 2].

## DISCUSSION

Preference for CAM in the study was reported by 26 (14.8%) of the study participants with absence of side effects found as its predictor. Alternative medicines are presumed to offer natural remedies for chronic conditions founded on its abilities to promote spirituality and enhance user’s connection to vitalism which has made its preference as adjunct to conventional treatments appealing to a number of people with these conditions.^[2]^ Preference for CAM was reported in less than a quarter of the respondents in study which is similar to what was also found in a study conducted in India though with focus on subjects with acute conditions. Furthermore, the same study reported much higher levels of preference for alternative medicines when chronic medical conditions were assessed.^[18]^ Contrary to the reported level of preference for CAM in the study, studies conducted in Kenya, Saudi Arabia, United States of America, Malaysia and Germany had slightly below half to most of their study participants having preference for CAM in the management of their medical conditions.^[17,19–22]^ The relatively low level of preference for CAM observed in this study could be attributable to the fact that a mixed population of subjects having different chronic medical conditions participated in this study as against in the other cited studies where patients with single chronic medical conditions were sampled. Additionally, environmental, socio-cultural and behavioural factors could have also contributed to the variation in the preference level documented in this study in relation to other studies cited. Preference for alternative medicines as complementary to the orthodox treatment implies that in the face poor treatment outcomes, clinicians should consider exploring the level of compliance with prescribed conventional treatment and assess the concomitant use of CAM as well as its possible contributions to unsatisfactory treatment outcomes. Furthermore, it is imperative that health care professionals periodically elicit information on the use or intention to use alternative therapies or medicines from their patients so as to ensure that holistic management of chronic medical conditions is provided. Health education and provision of information relevant to addressing treatments gaps and beliefs that may herald prioritizing the use of alternative medicines with prescribed treatments may also be required from attending physicians so as to achieve the expected treatment outcomes. Also, having identified that preference for alternative medicines is a common place among persons accessing long term conventional treatment for medical conditions, it is essential that other studies be conducted on the use of CAM among persons with chronic medical conditions in this setting to bring to bear the extent to which preference translates to utilization.Preference for CAM was found to be positively predicted by the perceived absence of side effects from its use in the study. However, diversity of factors such as sex, philosophically congruence, perception of better efficacy, affordability, peer/group influence, place of residence, availability, severity of illness, age, social stigma, treatability of the condition and perceived distress of medical treatment have been found to influence the level of preference for CAM from other studies.^[14,18,19]^ Importantly, if strict adherence to prescribed conventional medical treatment will be achieved and sustained, these factors would need to be focused on and addressed in the context of the needs of the individual patient along the chain of health care delivery for management of chronic conditions. This study has demonstrated some level of preference for CAM among patients on long term conventional treatment with perceived absence of side effects influencing this preference level.

## Figures and Tables

**Table 1:** Demographic characteristics and preference for Complementary and Alternative Medicine

Variables	Frequency (*n* = 176)	Percentage
Age (Years) ≤ 50	93	52.8
Females	108	61.4
Married	130	73.9
Duration of illness > 6 years and more	81	46.0
Urban residence	68	38.6
Christianity	134	76.1
Tertiary level of education	95	54.0
Employed in paid job	98	55.7
Co-morbidity present	48	27.3
Preference for CAM	26	14.8
Reasons for preference of CAM (*n* = 26)*		
Absence of side effects	24	92.3
Congruence with culture and beliefs	21	80.8
Affordability	14	53.9
Accessibility	22	84.6

* Multiple responses elicited.

**Table 2:** Predictor of preference for complementary and Alternative Medicine

Factors	COR (95%CI)	*P*-value	AOR (95% CI)	*P*-value
Age group (years)				
51 and above	0.95 (0.4138–2.1980)	0.911		
≤50				
**Sex**				
Male	1.20 (0.5140–2.7854)	0.677		
Female				
**Marital status**				
Single	1.09 (0.3374–3.5386)	0.882		
Separated/divorced	0.01 (0.0000 > 1.0E12)	0.971		
Widowed	0.40 (0.0495–3.2180)	0.389		
Married	1			
**Level of education**				
Primary	4.43 (0.3702–52.9840)	0.240		
Secondary	5.54 (0.6092–50.3041)	0.129		
Tertiary	7.35 (0.9271–56.6438)	0.059		
No formal education	1			
**Place of residence**				
Rural	1.81 (0.5996–5.4540)	0.293		
Urban	1.50 (0.5618–3.9814)	0.420		
Peri-urban	1			
**Current employment status**				
Unemployed	0.63 (0.2605–1.4825)	0.284		
Employed	1			
**Co-morbidity**				
Present	0.98 (0.3834–2.5024)	0.965		
Absent	1			
**Duration of illness (years)**				
6 and above	1.74 (0.7477–4.0286)	0.199		
≤5	1			
Perception of Side effects**				
Present	18.59 (12.7391–26.8824)	<0.001	11.34 (5.8299–15.1185)	<0.001
Absent			1	
**Congruence with culture and beliefs** *				
Yes	6.09 (2.2389–16.5557)	<0.004	1.33 (0.3102–5.6915)	0.702
No	1		1	
Affordability*				
Yes	3.73 (1.1400–12.2056)	0.030	2.54 (0.5436–11.9083)	0.232
No	1		1	
Accessibility*				
Yes	15.7 (5.6364–23.5461)	<0.001	2.56 (0.6246–10.5039)	0.191
No	1		1	

COR = Crude Odds Ratio, AOR = Adjusted Odds Ratio,

* Statistically significant before adjustment but not after adjustment.

** Statistically significant after adjustment.
